# Evolutionary conservation of P-selectin glycoprotein ligand-1 primary structure and function

**DOI:** 10.1186/1471-2148-7-166

**Published:** 2007-09-14

**Authors:** Bénédicte Baïsse, Frédérique Galisson, Sylvain Giraud, Marc Schapira, Olivier Spertini

**Affiliations:** 1 Service and Central Laboratory of Hematology, Centre Hospitalier Universitaire Vaudois, Bugnon 46, 1011 Lausanne, Switzerland; 2 Swiss Institute of Bioinformatics, Center for Integrative Genomics, UNIL, Dorigny, 1015 Lausanne, Switzerland

## Abstract

**Background:**

P-selectin glycoprotein ligand-1 (PSGL-1) plays a critical role in recruiting leukocytes in inflammatory lesions by mediating leukocyte rolling on selectins. Core-2 O-glycosylation of a *N*-terminal threonine and sulfation of at least one tyrosine residue of PSGL-1 are required for L- and P-selectin binding. Little information is available on the intra- and inter-species evolution of PSGL-1 primary structure. In addition, the evolutionary conservation of selectin binding site on PSGL-1 has not been previously examined in detail. Therefore, we performed multiple sequence alignment of PSGL-1 amino acid sequences of 14 mammals (human, chimpanzee, rhesus monkey, bovine, pig, rat, tree-shrew, bushbaby, mouse, bat, horse, cat, sheep and dog) and examined mammalian PSGL-1 interactions with human selectins.

**Results:**

A signal peptide was predicted in each sequence and a propeptide cleavage site was found in 9/14 species. PSGL-1 *N*-terminus is poorly conserved. However, each species exhibits at least one tyrosine sulfation site and, except in horse and dog, a T [D/E]PP [D/E] motif associated to the core-2 *O*-glycosylation of a *N*-terminal threonine. A mucin-like domain of 250–280 amino acids long was disclosed in all studied species. It lies between the conserved *N*-terminal *O*-glycosylated threonine (Thr-57 in human) and the transmembrane domain, and contains a central region exhibiting a variable number of decameric repeats (DR). Interspecies and intraspecies polymorphisms were observed. Transmembrane and cytoplasmic domain sequences are well conserved. The moesin binding residues that serve as adaptor between PSGL-1 and Syk, and are involved in regulating PSGL-1-dependent rolling on P-selectin are perfectly conserved in all analyzed mammalian sequences. Despite a poor conservation of PSGL-1 *N*-terminal sequence, CHO cells co-expressing human glycosyltransferases and human, bovine, pig or rat PSGL-1 efficiently rolled on human L- or P-selectin. By contrast, pig or rat neutrophils were much less efficiently recruited than human or bovine neutrophils on human selectins. Horse PSGL-1, glycosylated by human or equine glycosyltransferases, did not interact with P-selectin. In all five species, tyrosine sulfation of PSGL-1 was required for selectin binding.

**Conclusion:**

These observations show that PSGL-1 amino acid sequence of the transmembrane and cytoplasmic domains are well conserved and that, despite a poor conservation of PSGL-1 *N*-terminus, L- and P-selectin binding sites are evolutionary conserved. Functional assays reveal a critical role for post-translational modifications in regulating mammalian PSGL-1 interactions with selectins.

## Background

Leukocyte recruitment in inflammatory lesions is dependent on the sequential interactions of adhesion receptors with their ligands [1-3]. Leukocyte rolling along inflamed blood vessels is mediated by selectins [4-6]. L-selectin is expressed by leukocytes while activated endothelium and/or platelets express E- or P-selectin [5]. Early in inflammatory reactions [2,3], P-selectin mediates leukocyte rolling on its major ligand P-selectin glycoprotein ligand-1 (PSGL-1) [7]. PSGL-1 is a homodimeric mucin-like glycoprotein [8,9], which is expressed on leukocyte microvilli and functions as a common ligand for the three selectins [10,11]. PSGL-1 interactions with L-selectin strongly amplify leukocyte recruitment by supporting free-flowing leukocyte rolling on leukocytes adherent to microvascular endothelium or leukocyte membrane fragments [11,12]. Moreover, E-selectin interactions with PSGL-1 and CD44 and/or other potential ligands support leukocyte slow rolling along inflamed endothelium [13-15].

Fucosylated core-2 *O*-glycans, bearing sialyl Lewis-x (sLe^x^) and/or Le^x ^determinants, attached to human PSGL-1 Thr-57 are required for optimal binding of all three selectins [16-19]. Sulfation of Tyr-46, -48 and -51 is necessary for optimal binding of L- and P-selectin to PSGL-1 but not E-selectin [16-18,20-22]. Murine and human PSGL-1 may differ in their interactions with P-selectin, as sulfation of a single tyrosine residue is sufficient for optimal binding of murine PSGL-1 to P-selectin [23].

Human, mouse, rat, bovine and equine PSGL-1 sequences encode a signal peptide and, except for bovine and equine PSGL-1, a propeptide, which is predicted to be cleaved by paired basic amino acid converting enzymes (PACE) [9,24-26]. These sequences encode a common PSGL-1 primary structure with a *N*-terminal peptide expressing potentially sulfated tyrosine residues and a *O*-glycosylated threonine [9,24,25], and a mucin-like domain constituted of a variable number of decameric repeats (DR) [24-26]. Comparison of these mammal sequences shows that the transmembrane and cytoplasmic domains are highly conserved [9,27,28]. Little information is however available on the intra- and inter-species evolution of decameric motives and on the conservation of PSGL-1 *N*-terminus. Multiple sequence alignment of a large number of mammalian PSGL-1 sequences is necessary to examine these points and define motives associated with the core-2 *O*-glycosylation of the *N*-terminal threonine, homologous to Thr-57 on human PSGL-1.

Whether the selectin binding site on mammalian PSGL-1 is evolutionary conserved has not been studied in detail. As PSGL-1 is an attractive target for anti-inflammatory therapy [7,29-39], this information might be helpful to design inhibitors of inflammatory and/or thrombotic reactions [40-42]. We therefore compared PSGL-1 sequences of 14 mammals (9 sequences described herein by us and 5 reported by others; [9,24-26] and performed flow adhesion assays using neutrophils or CHO cells expressing mammalian homologues of human PSGL-1. Despite a poor conservation of the *N*-terminal amino-acid sequences, we show that L- and P-selectin binding sites are evolutionary conserved and that most mammalian PSGL-1 bind to human selectins. Importantly, these interactions are strongly dependent on PSGL-1 glycosylation and sulfation.

## Results

### Conservation of PSGL-1 sequence

**Figure 1:**
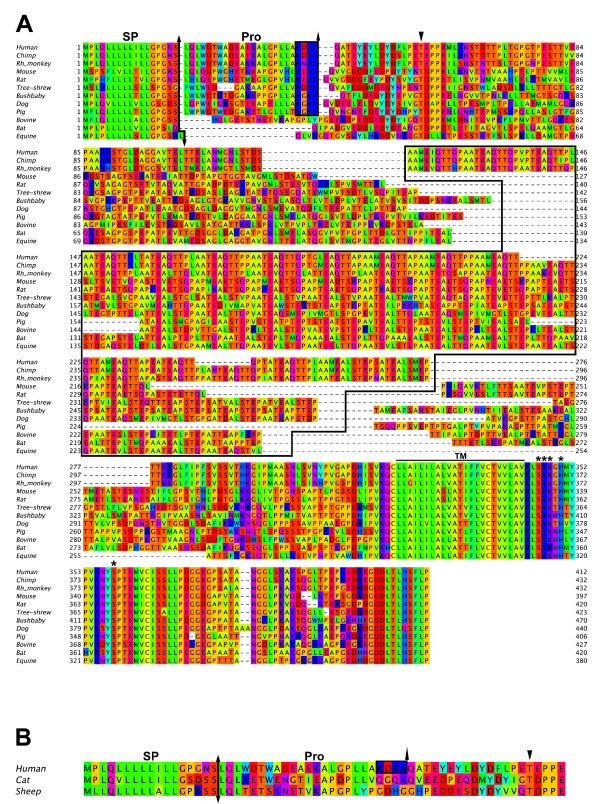
**Amino acid sequences of mammalian PSGL-1**. **(A) **Multiple alignment of 12 complete PSGL-1 amino acid sequences and **(B) **of *N*-terminal sequences from putative PSGL-1 proteins of cat and sheep, which were inferred respectively from partial genomic sequence and EST sequences identified through homology searches (EMBL/Genbank/DDBJ respective accession numbers: AANG01098304 and DY506895). The putative signal peptide (SP) and propeptide (Pro) cleavage sites are indicated by arrows. The consensus sequence for propeptide cleavage (RX [R/K]R), recognized by PACE, is boxed. Threonine homologous to human Thr-57 is indicated by a black arrowhead. Arbitrary gaps have been added in each sequence in order to isolate and align separately the mucin-like region containing the decameric repeats, which is surrounded by a frame. The transmembrane domain (TM) is marked by a bar. Asterisks indicate the amino acids involved in moesin binding.

Multiple alignment of mammalian PSGL-1 sequences is presented in figure 1. A signal peptide (SP) cleavage site is predicted between residues 17 and 18 in most sequences. Equine PSGL-1 is an exception with a predicted cleavage site between residues 18 and 19. Nine sequences including human have a propeptide predicted to be cleaved by paired basic amino acid-converting enzymes (PACE/furin; [9,43] at residue 41 (38 for northern tree shrew). By contrast, the PACE consensus sequence, RX [R/K]R is not observed in bovine, sheep, cat, bat and equine PSGL-1 (Fig. 1).

*N*-terminal tyrosine sulfate residues and threonine *O*-linked glycans are high affinity binding sites for P- and L-selectin to human and mouse PSGL-1 [16-18,20,44-46], which contribute to stabilize leukocyte rolling [16]. A threonine residue, homologous to human PSGL-1 Thr-57, is present in the various species studied here (Fig. 1). Thr-57 belongs to the consensus sequence T [D/E]PP [D/E] in 12 out of 14 species. The region preceding the conserved threonine contains 1 to 3 potentially sulfated tyrosine residues in an acid-rich region (5 species contain 3 tyrosines, 6 contain 2, and 3 only 1; Fig. 1).

A mucin-like domain is present in all studied species. It lies between the conserved *N*-terminal *O*-glycosylated threonine (Thr-57 in human) and the transmembrane domain, and contains a central region exhibiting decameric repeats (DR). This region was analyzed using the MEME program, whose parameters were applied to each sequence individually and/or simultaneously to all sequences. DR-containing central regions were aligned considering the intra- and inter-species evolution of decameric motives. The degree of inter-species conservation in the *N*- and *C*-terminal ends of the mucin-like domain (which sometimes contains traces of mutated decamers) is low. The mucin-like domain is composed of 247 to 322 residues and the number of DR varies from 7 in pig to 18 in chimpanzee and rhesus monkey (Table 2). The number of DR varies in human from 14 to 16 repeats [47-50]. We also observed a polymorphism in rat. One of the three available sequences contains 12 DR [51], whereas only 11 repeats have been observed in the sequences cloned by us (Fig. 1) and others [25]. This polymorphism suggests a dynamic intraspecies evolution of this region.

**Table 2:** Length of the mucin-like domain from the conserved threonine up to the juxta-membrane cysteine

	threonine position	cysteine position	length (aa)	number of decameric repeats
human	57	320	263	16
chimpanzee	57	340	283	18
rhesus monkey	57	340	283	18
mouse	58	307	249	10
rat	59	330	271	11
tree shrew	55	332	277	13
bushbaby	56	378	322	13
dog	59	346	287	13
pig	58	315	257	7
bovine	55	335	280	11
bat	37	328	291	12
equine	41	288	247	12

aa, amino acid

The analysis of the sequences of PSGL-1 mucin-like regions showed that several constitutive repeats of 10 amino acids can be identified in the center of these regions, while both ends are made up with unconserved amino acids. The best permutation motif, which is the most consistent with the different sequences and which optimizes the number of repeated units per sequence, is AATEAQTTQP.

Interestingly, in canine PSGL-1, 3 DR strongly differ in their sequences from the others (Fig. 1A). These units are identical to each other and are located every 30 positions. Combining decamera to form repeats of 30 amino acids displays a greater consensus between repeats suggesting that duplication of 30 amino acid units (itself created by two duplications of 10 amino acid units followed by mutations in the third one) arose at least twice in the evolution of dog PSGL-1. The same kind of phenomenon is observed in bat, where the best repeated unit has a length of 15 amino acids. Similarly, equine repeated units exhibit a greater consensus when they are formed of 20 residues units instead of 10 [52].

A transmembrane domain of 23 residues is predicted in all sequences immediately after the conserved cysteine involved in PSGL-1 dimerization (Fig. 1A). A short extracellular juxta-membrane region is involved in binding versican G3 domain, whose interaction with PSGL-1 promotes leukocyte aggregation [53]. Interestingly, three positions in this region are perfectly conserved in all studied species (Asp-313, Val-317, Lys-318). The transmembrane domain is followed by a cytoplasmic tail, which is made up of two highly conserved regions. Over the 31 first positions of the cytoplasmic domain, 20 are completely conserved and 5 contain conservative substitutions (Fig. 1A). Among the conserved positions, Ser-346, Arg-347, Lys-348 and Ser-358 (Fig. 1A) are involved in moesin binding to the cytoplasmic domain of human PSGL-1 [54]. In all sequences, the *C*-terminal region is ended by 11 almost perfectly conserved residues.

### Human L- and P-selectin interact with human, rat, bovine, pig or equine CHO-PSGL-1 cells

**Figure 2:**
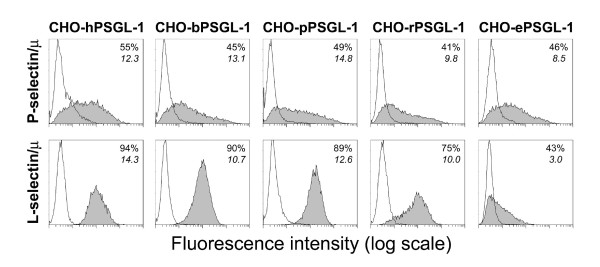
**Human L- and P-selectin/μ chimera cross-react with CHO cells expressing mammalian homologues of PSGL-1**. CHO cells, stably expressing similar levels of human C2GnT-I, FucT-VII and human (h), bovine (b), pig (p), rat (r) or equine (e) PSGL-1, were incubated with saturating concentrations of human P- or L-selectin/μ chimeras (filled histogram). Chimera binding was abrogated by 10 mM EDTA (open histogram). Human P- and L-selectin chimera did not bind (< 2%) to mock-transfected CHO cells (not shown). The percentage of positive cells and the mean fluorescence intensity are indicated in each histogram. Histograms are representative of 3–4 experiments.

CHO cells co-expressing human FucT-VII and C2GnT-I and human, bovine, pig, rat or equine PSGL-1 were prepared. The five transfectants expressed similar levels of sLe^x ^and CLA. PSGL-1 expression was detected using a mAb reacting with PSGL-1 *C*-terminal 6 × His tag (Invitrogen). The anti-human PSGL-1 mAbs PL1, KPL1 and PL2 [28,55] did not react with bovine, pig, rat or equine PSGL-1 (data not shown). Flow cytometric analysis of human P- or L-selectin/μ binding to the various CHO-PSGL-1 transfectants showed that P- and L-selectin/μ bind similarly to human, bovine, pig, rat or equine PSGL-1 expressed by transfected CHO cells. As the reactivity of mouse PSGL-1 with human selectins was previously described [23], we did not repeat these analyses (Fig 2).

### Human L-, P- and E-selectin bind heterogeneously to human, bovine, pig or rat neutrophils

**Figure 3:**
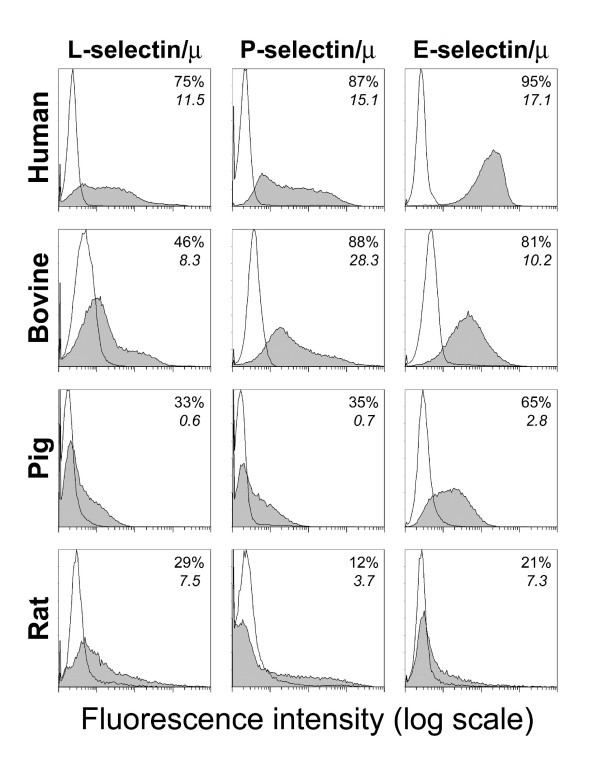
**Human L-, P- or E-selectin/μ chimeras bind to human, bovine, pig or rat neutrophils**. Neutrophils were incubated with saturating concentrations of human L-, P- or E-selectin/μ chimera (filled histogram). Chimera binding was abrogated by 10 mM EDTA (open histogram). The percentage of positive cells and the mean fluorescence intensity are indicated in each histogram. Histograms are representative of 2–3 experiments.

PSGL-1 expressed by CHO transfectants differ in their glycosylation pattern from mammalian neutrophil PSGL-1. In CHO transfectants, the various mammalian PSGL-1 are glycosylated by FucT-VII and C2GnT-I of human origin, while in mammalian neutrophils PSGL-1 is glycosylated by their own glycosyltransferases. As the glycosylation pattern may affect PSGL-1 interactions with L- or P-selectin, we examined the reactivity of human selectins with mammalian neutrophils (Fig. 3). L- and P-selectin/μ chimera strongly reacted with human and bovine PSGL-1, while a weaker reaction was observed with pig and rat. The L- and P-selectin carbohydrate ligands sLe^x ^and CLA, identified by CSLEX-1 and HECA-452 mAbs respectively, were strongly expressed by human neutrophils and also, surprisingly, by equine neutrophils (mean fluorescence intensity ± SD: human: 74 ± 1, n = 2 and 79 ± 12, n = 2; equine: 173 ± 9, n = 2 and 108 ± 34, n = 2). By contrast, despite significant selectin binding, sLe^x ^and CLA were undetectable on bovine, pig and rat neutrophils (not illustrated). As selectin binding is dependent on cell surface expression of fucosylated ligands, we examined FucT-VII mRNA expression by RT-PCR amplification of total RNA from bovine, pig, rat and equine neutrophils. FucT-VII mRNA transcripts were detected in all investigated species (data not shown). Thus, as previously established for mouse leukocytes [56], the lack of reactivity of mAbs CSLEX-1 and HECA-452 with most mammalian PSGL-1 is likely due to the strong specificity of these mAbs for human oligosaccharides. Moreover, the observation that mAbs CSLEX-1 and HECA-452 strongly react with equine neutrophils suggests that human and equine neutrophils exhibit common carbohydrate structures, which are not detectable in mouse, rat, pig or bovine.

### CHO cells expressing mammalian PSGL-1 efficiently roll on human L- or P-selectin

**Figure 4:**
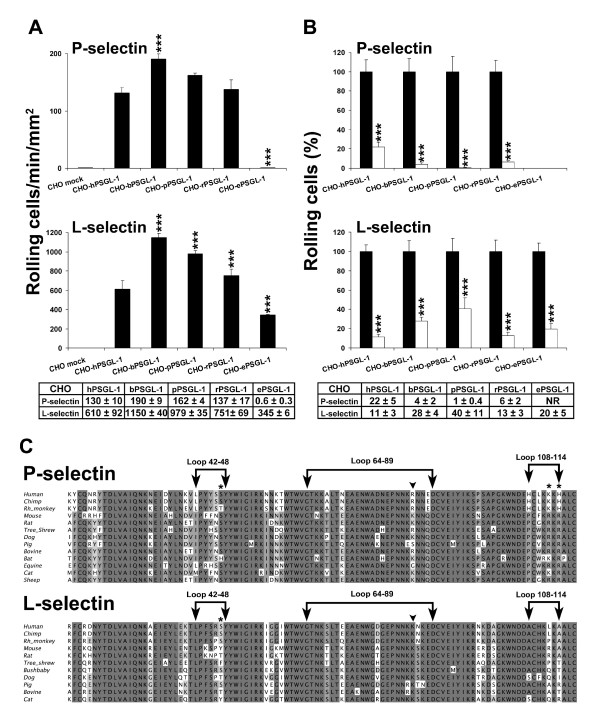
**Rolling of CHO cells expressing human, bovine, pig, rat or equine PSGL-1 on L- or P-selectin**. **(A) **CHO-PSGL-1 cells were perfused under constant shear stress (1.5 dynes/cm^2^) on recombinant human P-selectin or at 1.0 dyne/cm^2 ^on human L-selectin/μ chimera adsorbed on a coverslip, precoated with goat anti-human IgM antibody, and bound to the bottom of the flow chamber. Cell recruitment was analyzed by videomicroscopy at 4–5 min of perfusion. Results represent the mean ± SEM of 3–5 experiments (***, *P *< 0.001; NR: no rolling). **(B) **Impact of sulfation on PSGL-1-dependent rolling. Control (black columns) and desulfated CHO cells (white columns) were pretreated with proteinase K. Desulfated cells were cultured for 72 h in MEMα medium containing 60 mM sodium chlorate and exposed for 60 min to arylsulfatase. Results are expressed as mean percentage of rolling cells ± SEM of 3 experiments (***, *P *< 0.001). **(C) **Amino acid sequence alignments of mammalian homologues of P- and L-selectin lectin domains. Homologues of human residues [16, 18] interacting with sulfated Tyr-48 or -51 are respectively indicated by asterisks or arrowheads. The percentages of identity between aligned sequences are grey shaded (dark grey: > 80%, grey: > 60% and light grey: > 40%).

The role of PSGL-1 in regulating CHO-PSGL-1 cell rolling on human L- or P-selectin was assessed under hydrodynamic flow conditions. Human PSGL-1 expressing cells were less recruited on human P-selectin than CHO cells expressing bovine PSGL-1. Moreover, on human L-selectin, cell recruitment of CHO cells expressing human PSGL-1 was less efficient than that of cells expressing bovine, pig or rat PSGL-1 (Fig. 4A). Surprisingly, CHO cells expressing equine PSGL-1 did not roll on P-selectin and were weakly recruited on L-selectin.

Previous studies showed that *N*-terminal tyrosine sulfate residues are involved in supporting human PSGL-1-dependent rolling on L- and P-selectin [16,20,45]. Human, bovine, rat and pig PSGL-1 exhibit two or three potential *N*-terminal tyrosine sulfation sites, whereas equine PSGL-1 contains only one single site (Fig. 1A). The contribution of PSGL-1 sulfation to cell rolling was assessed by comparing recruitment of CHO cells expressing control or desulfated human, bovine, rat, pig and equine PSGL-1 on L- or P-selectin (Fig. 4B). Inhibition of PSGL-1 sulfation strongly reduced L- and P-selectin-dependent rolling. The recruitment of CHO cells expressing human PSGL-1, on P-selectin, was inhibited by 88 ± 5%, whereas the recruitment of cells expressing bovine, rat and porcine PSGL-1 was almost abrogated (Fig. 4B). Rolling inhibition induced by desulfation was also seen on L-selectin (although to a lesser degree than on P-selectin). Thus, as previously described for human PSGL-1, sulfation of bovine, pig, rat or equine PSGL-1 *N*-terminal tyrosine residues is required to support PSGL-1-dependent rolling on L- or P-selectin.

Interestingly, multiple sequence alignment of mammalian L- or P-selectin shows partial or complete conservation of amino acid residues that regulate human selectin binding to PSGL-1 tyrosine sulfate residues [16,18]. Ser-47, Lys-112 and His-114 on human P-selectin bind to human PSGL-1 Tyr-48, while human L-selectin Lys-85 and P-selectin Arg-85 interact with Tyr-51 (Fig. 4C) [16,18]. In mammalian P-selectins, Ser-47 is conserved, except for bat and rhesus monkey, and Lys-112 and His-114 is either conserved or replaced by arginine, which may interact with sulfated Tyr-48. Except for pig and horse, Arg-85, which binds to human PSGL-1 Tyr-51, is conserved or replaced by lysine (Fig. 4C). L-selectin Ser-47, which binds to human PSGL-1 Tyr-48, is conserved or replaced by a threonine, except for mouse, tree shrew and cat, while L-selectin Lys-85, which interacts with human PSGL-1 Tyr-51, is perfectly conserved. Results of Fig. 4B and alignment of Fig. 4C suggest that, like in human PSGL-1, tyrosine sulfation of mammalian homologues is critical for L- and P-selectin interactions. Sulfation of a unique tyrosine sulfate residue was sufficient to support equine PSGL-1-dependent rolling on human L-selectin (Fig. 4B). However, the recruitment of CHO cells expressing equine PSGL-1 on L-selectin was much less efficient than that of all other CHO cell transfectants (Fig. 4A).

### Mammalian neutrophil recruitment on human L- or P-selectin is heterogeneous

**Figure 5:**
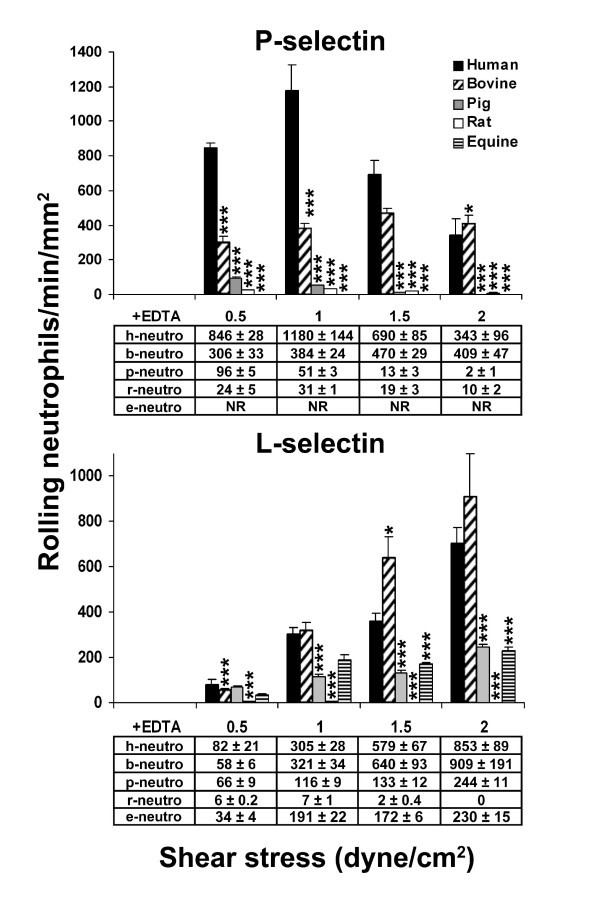
**L- and P-selectin-dependent recruitment of neutrophils is species-dependent**. Human, bovine, porcine, rat and equine neutrophils were perfused under 0.5–2.0 dynes/cm^2 ^on human P-selectin/μ or on human L-selectin/μ chimera adsorbed on coverslips precoated with goat anti-human IgM antibody. PSGL-1-dependent neutrophil rolling was abolished in presence of 10 mM EDTA. Cell recruitment was assessed at 4–5 min of perfusion. Results represent the mean ± SEM of 3–4 experiments (*, *P *< 0.05, ***, *P *< 0.001).

The impact of PSGL-1 glycosylation by mammalian FucT-VII and C2GnT-I on PSGL-1-dependent rolling on human L- or P-selectin was assessed under various shear stresses (0.5 to 2.0 dynes/cm^2^; Fig. 5). The recruitment of bovine, porcine, rat and equine neutrophils on human L- or P-selectin strongly differed from that of the corresponding CHO-PSGL-1 cells (Kruskal-Wallis test, *P *< 0.0001; Fig. 4A). At 1.5 and 2.0 dynes/cm^2^, bovine and human neutrophils rolled similarly on P-selectin. However, at lower shear stresses, bovine neutrophils were significantly less recruited than human neutrophils. Human and bovine neutrophil recruitment on L-selectin was similar at 1.0 and 2.0 dynes/cm^2 ^(Fig. 5). Above 0.5 dynes/cm^2^, porcine, rat and equine neutrophils were less recruited on L- or P-selectin than human or bovine neutrophils (Fig. 5). At 1.5 dynes/cm^2^, recruitment of porcine, rat and equine neutrophils was respectively 4-, 290- and 3-fold lower on L-selectin and 53- and 36-fold lower on P-selectin than that of human neutrophils. As observed with CHO cells expressing equine PSGL-1, equine neutrophils did not roll on P-selectin. At all shear stresses, rat neutrophils poorly rolled on human L- or P-selectin. These observations are in agreement with results of human selectin chimera binding to neutrophils (Fig. 3); both assays showed that bovine neutrophil PSGL-1 strongly interacts with human L- or P-selectin whereas interactions are weaker between human selectins and porcine neutrophil PSGL-1 and almost absent with rat and equine PSGL-1 (Fig. 5). These results are in contrast with those obtained with CHO cells expressing pig or rat PSGL-1, which are much more efficiently recruited on human selectins (Fig. 4A). Interspecies differences in PSGL-1 core-2 *O*-glycosylation may explain these observations.

### Rolling velocities of CHO-PSGL-1 transfectants and of mammalian neutrophils on human L- or P-selectin

**Figure 6:**
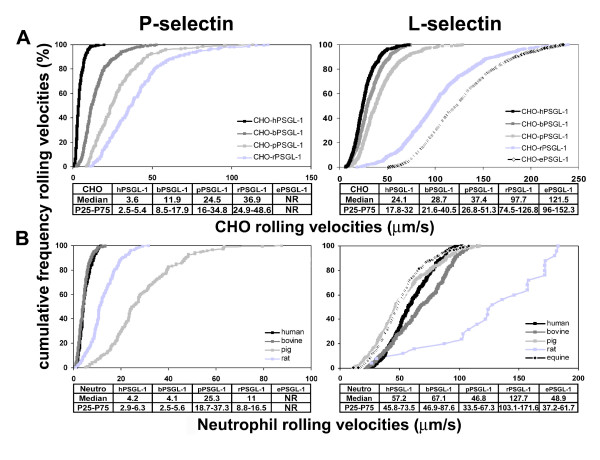
**Rolling velocities of CHO-PSGL-1 transfectants or neutrophils on human L- or P-selectin**. **(A) **CHO cells expressing human, bovine, pig, rat or equine PSGL-1 or **(B) **neutrophils were perfused under a constant shear stress on recombinant human P-selectin (1.5 dynes/cm^2^) or L-selectin/μ chimera (1.0 dyne/cm^2^). Cell velocities were measured at 4–5 min of perfusion. Curves were constructed in **(A) **using 183–755 or **(B) **25–381 independent determinations of cell-rolling velocities and are representative of three experiments. Median rolling velocities, representative of 3 experiments, are indicated.

Rolling velocities of CHO cells and neutrophils expressing human, bovine, pig, rat or equine PSGL-1 were measured under constant shear stress (Fig. 6). Velocities significantly differed among species (Fig. 6A, *P *< 0.001). CHO cells expressing human PSGL-1 rolled on P- or L-selectin with the slowest velocities (median rolling velocity (mrv) on P-selectin: 3.6 μm/s; on L-selectin: 24.1 μm/s, n = 3). The fastest mrv were exhibited by CHO cells expressing rat PSGL-1 on P-selectin (36.9 μm/s) and by CHO cells expressing equine PSGL-1 cells on L-selectin (121.5 μm/s). Mrv of CHO cells expressing bovine PSGL-1 on P-selectin appeared three times faster than that of CHO cells expressing human PSGL-1 (11.9 *vs*. 3.6 μm/s, *P *< 0.001), while they were similar on L-selectin (Fig. 6A). Rolling velocities of CHO cells expressing pig or rat PSGL-1 were significantly higher than that of CHO cells expressing human PSGL-1 on both L- and P-selectin (Fig. 6A, *P *< 0.001). Compared to CHO cells expressing human PSGL-1, increased velocities of CHO cells expressing bovine PSGL-1 on P-selectin may have resulted in increased cell recruitment on human selectins (Fig. 4).

The rolling velocities of human and bovine neutrophils on human P-selectin did not differ significantly (mrv: 4.2 μm/s *vs*. 4.1 μm/s, n = 3, Fig. 6B, left panel), whereas human neutrophils rolled slower on L-selectin than bovine neutrophils (57.2 μm/s *vs*. 67.1 μm/s, n = 3, *P *< 0.01, Fig. 6B, right panel). Surprisingly, porcine neutrophils rolled with the fastest velocities on human P-selectin (mrv: 25.3 μm/s, *P *< 0.001, n = 3; Fig. 6B, left panel), whereas they rolled, like equine neutrophils, with the slowest velocities on L-selectin (46.8 μm/s and 48.9 μm/s, respectively *P *< 0.001, Fig. 6B, right panel).

**Figure 7:**
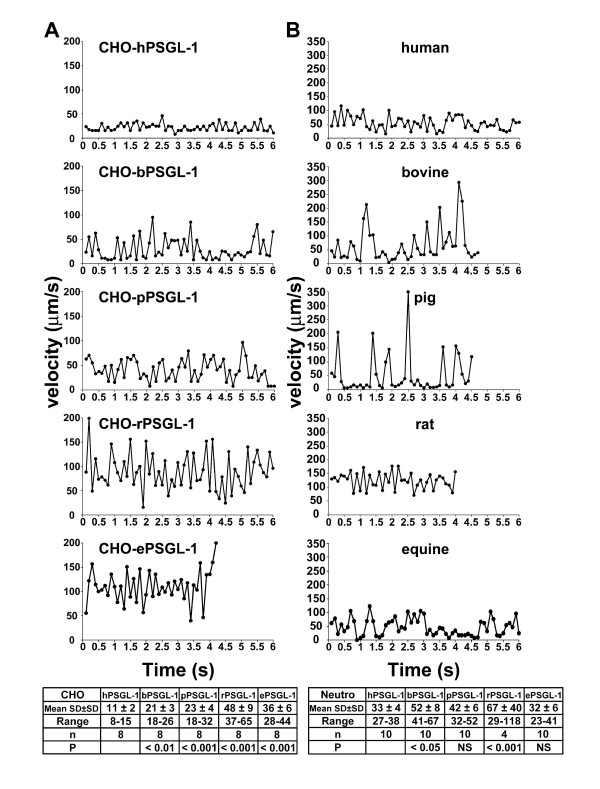
**Stability of rolling velocities of CHO-PSGL-1 transfectants or mammalian neutrophils on human L-selectin**. Frame-by-frame rolling velocities of (A) CHO-PSGL-1 transfectants or (B) human, bovine, pig, rat or equine neutrophils on human L-selectin. The velocity of tracked cells was determined by measuring cell displacements within successive video frames (0.1 ms) in the flow direction under a shear stress of 1.0 dyne/cm2. Cells were tracked for 4 to 6 s. Data are representative of 4–10 experiments.

The stability of rolling velocities was assessed by measuring CHO-PSGL-1 cell and neutrophil displacements on human L-selectin within successive video frames (0.1 ms). Peaks represent increases in velocity and valleys decreases (Fig. 7). The stability of CHO-PSGL-1 cell rolling velocities on human L-selectin was heterogeneous among the studied species. Although CHO cells expressing human and bovine PSGL-1 had similar mrv, rolling velocities of CHO cells expressing bovine PSGL-1 (mean SD ± SD: 21 ± 3 μm/s) were less stable than those of cells expressing human PSGL-1 (11 ± 2 μm/s). CHO cells expressing rat PSGL-1 were the least stable (48 ± 9 μm/s, Fig. 7A). The stability of neutrophil rolling velocities was also highly heterogeneous among the studied species (Fig. 7B). Human and equine neutrophils exhibited the most stable rolling velocities (mean SD ± SD: 33 ± 4 μm/s *vs*. 32 ± 6 μm/s, n = 10, NS, Fig. 7B), whereas rat neutrophils were the least stable (67 ± 40 μm/s, n = 4). Interestingly, pig neutrophils exhibited periods of very slow rolling (mean velocity < 10 μm/s) alternating with sudden acceleration, rapidly followed by deceleration (Fig. 7B). Bovine and equine neutrophils had similar behaviors. Despite the presence of oligosaccharides recognized by HECA-452 and CSLEX-1 mAbs on both CHO cells and neutrophils expressing equine PSGL-1, transfected CHO cells rolled significantly faster and less stably than equine neutrophils, suggesting that other structures regulate equine neutrophil rolling.

## Discussion

Selectins and PSGL-1 play a critical role in regulating leukocyte migration in inflammatory lesions [4]. Whether human selectins can bind to mammalian PSGL-1 had not been previously studied. As PSGL-1 is an attractive target for anti-inflammatory therapy [29-33,35,37-39], the identification of conserved PSGL-1 functional regions may be helpful to design selectin inhibitors mimicking PSGL-1. We therefore analyzed PSGL-1 amino acid sequences of several mammals (5 previously described sequences; [9,24-26] as well as 9 new sequences described herein, 7 complete and 2 *N*-terminal sequences; Fig. 1A–B) identified by us or others [25,26]. Multiple sequence alignments (Fig. 1) show that conservation of sequence is not homogeneous along the protein, and that the primary sequence of the site of interaction of L- or P-selectin [16,18] is not perfectly conserved. All sequences contain a threonine homologous to the core-2 *O*-glycosylated Thr-57 in human, and a T [D/E]PP [D/E] motif, which is conserved in all species, except in horse and dog. Nevertheless, even if the region preceding this threonine always contains at least one tyrosine residue in an anionic environment (predicting sulfation; [57] its length in the mature protein, as well as the number (1 to 3) and positions of the potentially sulfated tyrosine residues are variable. Desulfation and sulfation inhibition studies suggest that tyrosine sulfation plays a key role in supporting mammalian PSGL-1 interaction with human L- and P-selectin (Fig. 4B). Data presented here indicate that L- and P-selectin binding sites on PSGL-1 are evolutionary conserved and emphasize the role of threonine-linked core-2 *O*-glycans and tyrosine sulfate residues in supporting mammalian PSGL-1 interactions with human selectins.

A signal peptide is predicted to be cleaved in all PSGL-1 sequences between positions 17 and 18, except in horse where cleavage is predicted between Leu-18 and Gln-19. Nine out of fourteen sequences exhibit a propeptide sequence ended by a PACE cleavage site, whereas five others (horse, bat, bovine, cat and sheep) do not contain it. Of note, the cleavage predictions of both the signal peptide and the propeptide have been corroborated in rat by *N*-terminal sequencing of PSGL-1.[25] Cleavage predictions suggest that the mature PSGL-1 protein starts at position 42 of the precursor in most studied species, but at position 18 or 19 in five other species (bovine, bat, horse, cat, sheep), and that the length of the *N*-terminal sequence preceding the *O*-glycosylated threonine varies from 14 amino acids in bushbaby to 39 in cat and sheep.

PACE cleaves PSGL-1 propeptide on human neutrophils. In contrast, the propeptide remains on CHO-PSGL-1 cells, which do not express the PACE protease. The lack of PSGL-1 cleavage by PACE in bovine and equine neutrophils did not prevent PSGL-1 interactions with selectins. The importance of propeptide cleavage is unclear: whether it may facilitate tyrosine sulfation or *N*-terminal *O*-glycosylation remains to be determined [21].

The T [D/E]PP [D/E] sequence, which is associated on human and mouse PSGL-1 with threonine *O*-glycosylation [9,18,58], is observed in most mammals except dog and horse, in which it is respectively replaced by TDAPE and TDLLK sequences. Despite these changes, equine neutrophils rolled on human L-selectin (Fig. 5). By contrast, neither equine neutrophils nor CHO cells expressing equine PSGL-1 significantly interacted with human P-selectin (Fig. 4, 5). This suggests that the T [D/E]PP [D/E] motif may be important for mammalian leukocyte rolling on human P-selectin.

We observed that the sequence AATEAQTTQP is the best permutation motif to optimize the number of decameric units per sequence and that the most similar units lie at the center of the mucin-like region, while unconserved amino acids are more frequently present at both ends. This suggests that decamera located at the center of the mucin-like domain might be the most recent and that the evolution of this region might have proceeded by duplications of decameric units, followed by mutations and deletions. This process allowed the conservation of the length of the mucin-like domain with a 250–280 amino acid length (except in bushbaby), despite a variable number of repeated units among species (from 7 DR in pig to 18 DR in monkeys, Table 2). The preservation of PSGL-1 length may play a role in supporting the rolling on human selectins of leukocytes or CHO cells expressing human, bovine, pig or rat PSGL-1 (Fig. 4).

Transmembrane and cytoplasmic domain sequences are well conserved (Fig. 1). The juxta-membranous cysteine residue, involved in human PSGL-1 dimerization and in stabilizing leukocyte rolling on P-selectin [27,28,59] is perfectly conserved. A role for PSGL-1 as signaling molecule was indicated by its involvement in activating GTPase Ras and mitogen-activated protein kinases, as well as in inducing the secretion of inflammatory molecules [60-62] or in activating αMβ_2 _or αLβ_2 _integrins [63-65]. The high degree of conservation of the cytoplasmic domain suggests that PSGL-1-mediated intracellular signaling is evolutionary conserved. Human PSGL-1 engagement induces Syk phosphorylation and recruitment in lipid rafts as well as the expression of the early-immediate gene *c-fos *[54,66]. Syk activity, which is critically involved in regulating PSGL-1-dependent rolling on P-selectin [66], is dependent on the binding of PSGL-1 cytoplasmic domain to moesin, which serves as adaptor between PSGL-1 and Syk [54]. Importantly, the moesin binding residues, corresponding to Ser-346, Arg-347, Lys-348, and Ser-358 in human PSGL-1 [54] are perfectly conserved in all analyzed mammalian sequences. Of note, these amino acids are located within a group of 31 amino acids, among which 20 are identical and 5 similar.

On L-selectin, rolling velocities of CHO cells expressing human, bovine, and pig PSGL-1 were similar, whereas the median rolling velocity of CHO cells expressing rat or equine PSGL-1 was 4- and 5-fold higher respectively than that of CHO cells expressing human PSGL-1 (Fig. 6). The increased rolling velocities of CHO cells expressing bovine, pig or rat PSGL-1 on P-selectin may partially explain the preserved cell recruitment on P-selectin (Fig. 4A). As all CHO-PSGL-1 transfectants are glycosylated by human C2GnT-I and FucT-VII, differences in CHO-PSGL-1 cell recruitment and rolling velocities may mainly result from differences in *N*-terminal amino-acid residues interacting with the lectin domain of human L- or P-selectin. Among these residues, tyrosine sulfate residues may critically regulate PSGL-1 interactions with L- or P-selectin, like in human PSGL-1 [16,18,22,45]. The strong inhibition of CHO-PSGL-1 cell interactions with P- or L-selectin after desulfation and inhibition of sulfation supports this possibility (Fig. 4B). In addition, in most studied mammals, the amino acids regulating selectin interactions with potentially sulfated tyrosine residues are conserved (Fig. 4C). In mouse, Tyr-54 and Thr-58 regulate PSGL-1 interactions with P-selectin.[23] Because only one tyrosine is used, it was suggested that mouse PSGL-1 binding may rely more on *O*-glycans attached to Thr-58 than does human PSGL-1.[23] This may also occur in other mammals, which exhibit a single tyrosine residue (tree shrew, bat and horse, Fig. 1).

Differences in tyrosine sulfation and *O*-glycosylation may affect the stability of rolling velocities on L-selectin. Thus, the patterns of bovine, pig and equine neutrophil displacements differed from those of CHO cells expressing mammalian PSGL-1. In particular, pig neutrophils, and also bovine and equine neutrophils, exhibited periods of very slow rolling velocity, alternating with rapid accelerations and decelerations (Fig. 7). These observations emphasize the role of post-translational modifications in regulating PSGL-1 binding to human selectins.

## Conclusion

Data presented here indicate that mammalian PSGL-1 share a common primary structure and has evolutionary conserved interactions with L- and P-selectin. As in human, PSGL-1-dependent rolling is regulated by core-2 *O*-glycosylation of a conserved threonine residue and by tyrosine sulfation. The high degree of conservation of PSGL-1 cytoplasmic domain suggests, as for human PSGL-1, a potential involvement in signal transduction and in regulating cell rolling. These results provide additional insights into the structure and function of PSGL-1 and may be helpful to design PSGL-1 peptidomimetics.

## Methods

### Bovine, porcine, murine and equine PSGL-1 and FucT-VII cDNAs

RNA was extracted from mammalian lymphocytes using TRIzol^® ^(Invitrogen, Basel, Switzerland). Bovine, pig and rat homologues of human PSGL-1 cDNAs were generated from lymphocyte total RNA using GeneRacer™ Kit (Invitrogen), according to the manufacturer protocols. Primer design was based on sequence homologies between human and mouse PSGL-1 [9,24]. Primers are listed in Table 1.

**Table 1:** Sequences of primers used for RACE and RT-PCR analysis

primer name	Sequence	Ta
GeneRacer™ 5'	5'-CGACTGGAGCACGAGGACACTGA-3'	64°C
Reverse GSP	5'-CAGACCATCTCGGTGGGGGAGTA-3'	
GeneRacer™ 5' Nested	5'-GGACACTGACATGGACTGAAGGAGTA-3'	55°C
Reverse Nested GSP	5'-CACAGTGCACACGAAGAAGATAGTG-3'	
GeneRacer™ 3'	5'GCTGTCAACGATACGCTACGTAACG-3'	55°C
Forward GSP	5'-ACTCCACTGGCAGCCACAGAGG-3'	
GeneRacer™ 3' Nested	5'CGCTACGTAACGGCATGACAGTG-3'	56°C
Forward Nested GSP bovine	5'-CCCTTCCTGTGGCCTCTGATACTC-3'	
Forward Nested GSP pig	5'-ACCAGCACCCACGGAGGCACAGACC-3'	
Forward Nested GSP rat	5'-CCCTGCCAGGGAGTTCAGATCTC-3'	
Forward hPSGL-1/AflII	5'-AGCCTTAAGCCACCATGCCTCTGCAACTCC-3'	54°C
Forward b, pPSGL-1/AflII	5'-TATCTTAAGCCACCATGTTTCTGCAACTCC-3'	
Forward rPSGL-1/AflII	5'-CGCCTTAAGCCACCATGTTCCCACACT-3'	
Forward ePSGL-1/AflII	5'-AGCCTTAAGCCACCATGCCTCTGCCGCTC-3'	
Reverse h, b, p, r, ePSGL-1/AgeI/***ClaI***	5'-TGGACCGGT***ATCGAT***AGGGAGGAAGCTGTG-3'	
Forward h, b, p, rFucT-VII	5'-TCCTTGTCTGGCACTGG-3'	50°C
Reverse h, b, p, rFucT-VII	5'-GCGGTGCTGGGAGTTCT-3'	
Forward h, b, p, rβ-actin	5'-GAGACCTTCAACACCCC-3'	50°C
Reverse h, b, p, rβ-actin	5'-GTGGTGGTGAAGCTGTAGCC-3'	

Ta, annealing temperature; GSP, gene specific primer; h, human; b, bovine; p, pig; r, rat; e, equine; FucT-VII, α1–3 fucosyltranferase-VII.

Full-length PSGL-1 cDNAs were obtained using primers specific for each species (Table 1): forward human, bovine, pig, rat and equine PSGL-1 contain an AflII restriction site and reverse PSGL-1 primers an AgeI and a ClaI restriction site removing the stop codon. Forty amplification cycles were performed using the Platinum^® ^Pfx DNA Polymerase (Invitrogen; 30 s at 94°C, 45 s at 54°C, 2 min at 72°C). PCR products were gel-purified, sequenced, digested with AflII/AgeI and cloned in the pcDNA5/FRT/V5-His-TOPO^® ^expression vector containing, C-terminally, 6 × His tag (Invitrogen).

α1–3 fucosyltranferase-VII (FucT-VII) mRNAs from human, bovine, pig, rat and equine neutrophils were amplified using the Superscript™ One-Step RT-PCR with platinum^® ^Taq Kit (Invitrogen). Primers were derived from human and mammalian FucT-VII sequences (Table 1). β-actin transcripts were used as control.

### Cells

Mammalian lymphocytes were isolated by blood centrifugation on Ficoll and polymorphonuclear cells (PMN) were obtained by dextran sedimentation and erythrocyte hypotonic lysis [10]. Flp-In™-CHO-K1 cells (Invitrogen) stably expressing core2 β(1,6)-*N*-acetyglucosaminyltransferase-I (C2GnT-I) and FucT-VII [16] were transfected using TransIT^®^-LT1 (Mirus Corporation, Madison, WI) with human, bovine, pig, equine or rat PSGL-1 constructs. CHO cells were cultured in MEMα medium (Invitrogen) containing 10% fetal calf serum (FCS), 800 μg/mL G418 (Invitrogen) and 700 μg/mL Hygromycin B (Calbiochem-Novabiochem, Schwalbach, Germany). CHO cells co-expressing similar levels of sialyl Lewis × (sLe^x^), cutaneous lymphocyte antigen (CLA) and PSGL-1 terminated by C-terminal polyhistidine (6 × His) tag were isolated by limiting dilution. CHO-P-selectin and 300.19-L-selectin cells were cultured as described [19].

### Inhibition of sulfation

CHO-PSGL-1 cells (10^7 ^cells in 1 mL of PBS) were treated with proteinase K (170 μg/mL; Roche Diagnostics, Rotkreuz, Switzerland) for 20 min at 37°C.[67] After proteinase K inhibition with phenylmethylsulphonylfluoride (Sigma-Aldrich, St-Louis, USA), cells were cultured for 72 h in sulfate-deficient MEMα medium containing 10% dialyzed FCS and 60 mM sodium chlorate (Sigma [68]). They were then further desulfated, for 60 min at 37°C, with *Aerobacter aerogenes *arylsulfatase (1 U/ml in PBS, type VI, Sigma).

### Immunophenotypic analysis

Cell staining with mAbs or L-, P-, or E-selectin/IgM heavy chain (μ) chimera was performed and analyzed with a Cytomics™ FC 500 cytofluorimeter (Beckman Instruments, Nyon, Switzerland), as described [19].

### Flow adhesion assays

Cells (10^6^/mL) were perfused in a parallel plate flow chamber (GlycoTech Corp., Rockville, MD) mounted on a glass coverslip covered with a confluent monolayer of CHO cells or coated with L-selectin/μ (2.0 μg in 100 μL 0.1 M borate buffer, pH 8.5, surface: 75 mm^2^) or P-selectin/μ (0.1 μg in 100 μL borate buffer) chimera or recombinant P-selectin (0.5 μg in 100 μL borate buffer) (R&D Systems, Minneapolis, MN) adsorbed on goat anti-human IgM antibody (2.0 μg in 100 μL 0.1 M borate buffer, pH 8.5; Caltag Laboratories, Burlingame, USA; [16,19,69]. CHO-PSGL-1 cell and neutrophil interactions were recorded for 5 min by videomicroscopy [16,19,69]. Rolling velocities illustrated in Fig. 6 were measured by tracking individual cells every 0.1 s, for 1–20 s, using a digital image analysis system (Mikado software, GPIL SA, Martigny, Switzerland; [16,19,69]. 183–755 independent determinations of cell rolling velocities were measured to analyze the velocities of transfectants and 25–381 determinations for the analysis of neutrophil velocities. Frame-by-frame velocities (Fig. 7) were measured by tracking cells every 0.1 s for 6 s, within 0.28 mm^2 ^microscopic fields. The mean velocity of frame-by-frame tracked cells was included between percentiles 40–60 of the velocity of each cell population illustrated in Fig. 6. L- and P-selectin-dependent rolling was inhibited (>95%) by 10 mM EDTA or LAM1–3 or WAPS12.2 mAbs (data not shown). Mock-transfected CHO cells did not roll on L- or P-selectin. CHO transfectants used in adhesion assays expressed similar levels of cell surface PSGL-1 and sLe^x ^[19].

### Sequences

PSGL-1 amino-acid sequences were either retrieved from the Uniprot database [70], or deduced from our own cDNA sequences (bovine, equine, pig and rat respective accession numbers [EMBL: AM778464, AM778465, AM778466, AM778467]), or inferred from their gene sequences identified through homology searches (chimpanzee, rhesus monkey, dog, bat, northern tree shrew, and bushbaby respective EMBL/Genbank/DDBJ accession numbers: AADA01122192, AANU01210210, AAEX02034222, AAPE01064070, AAPY01200400, and AAQR01577322).

Most selectin sequences were retrieved from Uniprot. Accession numbers of human, chimpanzee, rhesus monkey, rat, mouse and bovine L-selectins are P14151, Q95237, Q95198, Q63762, P18337 and P98131, respectively. Those of human, rat, mouse, bovine, dog, pig, equine and sheep P-selectin are P16109, P98106, Q01102, P42201, Q28290, Q29097, Q5J3Q6 and P98109, respectively. The chimpanzee and rhesus monkey P-selectin sequences were retrieved from the Refseq database^49 ^(IDs: XM_001137240 and XM_001094728). Dog, pig, northern tree shrew, bushbaby, cat L-selectins, and bat, northern tree shrew and cat P-selectins were predicted from their DNA sequences (EMBL/Genbank/DDBJ respective accession numbers: AAEX02026138, BW973806, AAPY01338510, AAQR01653637, AANG01023786, AAPE01015496, AAPY01338519, AANG01023773).

### Sequence analysis

Multiple alignments were obtained by analyzing local and global similarities between PSGL-1 (Fig. 1) or selectin sequences (Fig. 4) using Clustal-W, T-Coffee and MEME programs [71-73]. Alignment was edited and colored using the Jalview program [74]. Signal peptides, propeptides and transmembrane domains were predicted with the SignalP, ProP, and TMHMM programs [75-77].

### Statistical Analysis

Analysis of variance and the Bonferroni multiple comparison test or the Kruskal-Wallis non-parametric ANOVA test were used to assess statistical significance of differences between groups. The Mann-Whitney test was used to compare the medians of two unpaired groups. *P *values < 0.05 were considered as significant.

## Abbreviations

CHO cells - Chinese Hamster Ovary cells;

DR - Decameric repeats; FucT, fucosyltransferases; 

PACE - Paired basic amino acid converting enzymes; 

PSGL-1 - P-selectin glycoprotein ligand-1; 

sLe^x^ - Sialyl Lewis-x; 

TM - Transmembrane domain; 

mrv - Median rolling velocity.

## Authors' contributions

OS, BB and FG designed research, analyzed data and wrote the paper. BB and SG performed experiments. FG performed multiple alignment. MS critically reviewed the manuscript and contributed to writing. All authors read and approved the final manuscript
